# Wide spread and diversity of mutation in the *gyrA* gene of quinolone-resistant *Corynebacterium striatum* strains isolated from three tertiary hospitals in China

**DOI:** 10.1186/s12941-021-00477-0

**Published:** 2021-10-01

**Authors:** Yingjun Wang, Xiaohong Shi, Jian Zhang, Yanyan Wang, Yingying Lv, Xiaoli Du, QiQiGe ChaoLuMen, Junrui Wang

**Affiliations:** 1 Department of Laboratory Medicine, Affiliated Hospital of Inner Mongolian Medical University, 010050 Hohhot, People’s Republic of China; 2 Department of Clinical Laboratory Medicine, The First Affiliated Hospital of Shandong First Medical University & Shandong Provincial Qianfoshan Hospital, Shandong Medicine and Health Key Laboratory of Laboratory Medicine, 250014 Jinan, People’s Republic of China; 3 Department of Laboratory Medicine, Bayannur People’s Hospital, 015000 Bayannur, People’s Republic of China; 4 National Institute for Communicable Disease Control and Prevention, Chinese Center for Disease Control and Prevention, 102206 Beijing, People’s Republic of China; 5 Pediatric Ward, Affiliated Hospital of Inner Mongolian Medical University, 010050 Hohhot, People’s Republic of China

**Keywords:** *Corynebacterium striatum*, Multi-drug resistance, Genotyping, Quinolone resistance, Nosocomial outbreak

## Abstract

**Background:**

*Corynebacterium striatum* was confirmed to be an important opportunistic pathogen, which could lead to multiple-site infections and presented high prevalence of multidrug resistance, particularly to quinolone antibiotics. This study aimed to investigate the mechanism underlying resistance to quinolones and the epidemiological features of 410 quinolone-resistant *C. striatum* clinical strains isolated from three tertiary hospitals in China.

**Methods:**

A total of 410 *C. striatum* clinical strains were isolated from different clinical samples of patients admitted to three tertiary teaching hospitals in China. Antibiotic susceptibility testing was performed using the microdilution broth method and pulsed-field gel electrophoresis (PFGE) was used for genotyping. Gene sequencing was used to identify possible mutations in the quinolone resistance-determining regions (QRDRs) of *gyrA*.

**Results:**

In total, 410 *C. striatum* isolates were sensitive to vancomycin, linezolid, and daptomycin but resistant to ciprofloxacin. Depending on the antibiotic susceptibility testing results of 12 antimicrobial agents, the 410 *C. striatum* strains were classified into 12 resistant biotypes; of these, the three biotypes R1, R2, and R3 were dominant and accounted for 47.3% (194/410), 21.0% (86/410), and 23.2% (95/410) of the resistant biotypes, respectively. Mutations in the QRDRs of*gyrA* were detected in all quinolone-resistant *C. striatum* isolates, and 97.3% of the isolates (399/410) showed double mutations in codons 87 and 91 of the QRDRs of *gyrA*. Ser-87 to Phe-87 and Asp-91 to Ala-91 double mutation in *C. striatum* was the most prevalent and accounted for 72.2% (296/410) of all mutations. Four new mutations in *gyrA* were identified in this study; these included Ser-87 to Tyr-87 and Asp-91 to Ala-91 (double mutation, 101 isolates); Ser-87 to Val-87 and Asp-91 toGly-91 (double mutation, one isolate); Ser-87 to Val-87 and Asp-91 to Ala-91 (double mutation, one isolate); and Ser-87 to Ile-87 (single mutation, one isolate). The minimum inhibitory concentration of ciprofloxacin for isolates with double (96.5%; 385/399) and single (72.7%; 8/11) mutations was high (≥ 32 µg/mL). Based on the PFGE typing results, 101 randomly selected *C. striatum* strains were classified into 50 genotypes (T01-T50), including the three multidrug-resistant epidemic clones T02, T06, and T28; these accounted for 14.9% (15/101), 5.9% (6/101), and 11.9% (12/101) of all genotypes, respectively. The multidrug-resistant T02 clone was identified in hospitals A and C and persisted from 2016 to 2018. Three outbreaks resulting from the T02, T06, and T28 clones were observed among intensive care unit (ICU) patients in hospital C between April and May 2019.

**Conclusions:**

Quinolone-resistant *C. striatum* isolates showed a high prevalence of multidrug resistance. Point mutations in the QRDRs of *gyrA* conferred quinolone resistance to *C. striatum*, and several mutations in *gyrA* were newly found in this study. The great clonal diversity, high-level quinolone resistance and increased prevalence among patients susceptible to *C. striatum* isolates deserve more attention in the future. Moreover, more thorough investigation of the relationship between quinolone exposure and resistance evolution in *C. striatum* is necessary.

## Background

Recently, several reports have revealed that *Corynebacterium striatum* leads to multiple invasive infections [1, 2], and most *C. striatum* isolates are multidrug resistant, particularly to quinolones [3]. Point mutations in codons 87 and 91 in the quinolone resistance-determining regions (QRDRs) of *gyrA* were believed to be majorly responsible for the resistance of *C. striatum* to quinolones [4]. Quinolones tend to accumulate in the organs, and the resistant subpopulations of some common genus of bacteria may be selected upon exposure to quinolones, including multiple kinds of bacteria that colonize the skin and mucous membranes (such as corynebacteria) [5].

Although a high frequency of *C. striatum* was recently reported in China, the mechanism underlying the resistance of *C. striatum* to quinolones has been rarely reported in China [6, 7]. In addition, quinolone consumption was observed to be high in many hospitals in the last decade [8]. Furthermore, the previous use of fluoroquinolones or beta-lactam antibiotics was believed to be an essential risk factor for promoting the colonization or infection of *C. striatum* [9]. *C. striatum* is known to colonize multiple environmental and bodily surfaces [10] and spread among susceptible patients or even lead to outbreaks, which were observed in previous studies [3, 11].

This study investigated the actual resistance mechanism of *C. striatum* to quinolones with a larger number of isolates collected from three tertiary hospitals in China and explored its genotypic characterization and prevalence potential.

## Methods

### Isolation and identification of *C. striatum*

The hospitals enrolled in this study included The Affiliated Hospital of Inner Mongolian Medical University (hospital A, 3,000 beds), Shandong Provincial QianFuoShan Hospital (hospital B, 2,813 beds), and Bayannaoer People’s Hospital (hospital C, 1,700 beds), China, from March 2013 to May 2019. All *C. striatum* strains isolated from aseptic sites were identified. The quality of the sputum samples was evaluated for qualification based on the number of leukocytes and epithelium via microscopy [3], and the isolates collected from the qualified samples were enrolled in this study. All cultures suspected to be positive for *C. striatum* were routinely identified using VITEK-2 ANC card (BioMérieux, France) and stored at − 80 °C. The isolates were further validated by MALDI-TOF microTyper (Tianrui, China) as well as *16S rRNA* and *rpoB* sequencing technique [12]. Only one *C. striatum* strain from the same patient was selected in this study, whereas the repeated ones were excluded.

### Antibiotic susceptibility test

The antibiotic susceptibility test was performed using the broth microdilution method, and the antibiotics tested include penicillin (1–64 μg/mL), cefepime (1–64 μg/mL), imipenem (1–64 μg/mL), linezolid (0.5–4 μg/mL), erythromycin (0.5–64 μg/mL), clindamycin (1–32 μg/mL), gentamycin (1–32 μg/mL), tetracycline (1–64 μg/mL), vancomycin (0.5–4 μg/mL), sulfamethoxazole (0.5/9.5–8/152 μg/mL), daptomycin (0.06–1 μg/mL), ciprofloxacin (1–256 μg/mL), and moxifloxacin (0.06–32 μg/mL). The susceptibility test and result analysis were performed according to the 30th edition of the Clinical and Laboratory Standards Institute guidelines [13] and the recommendation of the European Committee on Antimicrobial Susceptibility Testing [14]. *Streptococcus pneumoniae* ATCC 49619 was used as the control.

### Pulsed-field gel electrophoresis (PFGE)

The bacterial density of the tested *C. striatum* isolates was adjusted to 3.5–4.0 Mcf and digested with lysostaphin (1 mg/ml) (Merck, USA) at 37 °C for 30 min. The bacterial chromosomal DNA of the isolates was extracted and cleaved using 40 U SwaI (Takara, China). The DNA of the *S. Braenderup* H9812 standard strain was extracted and cleaved using 40 U XbaI (Takara, China) and utilized as the molecular mass standard. Electrophoresis was performed on the CHEF-Mapper XA PFGE system (Bio-Rad, Hercules, CA, USA), and PFGE profiles were analyzed using the Bionumerics v.7.6 software. The isolates with 100% similarity were considered indistinguishable [15, 16], and each clone was named using a single capital letter.

### Detection of mutation in the QRDR region of *gyrA*

The bacterial chromosomal DNA of the tested *S. aureus* isolates was extracted as per the instructions of the TIANamp Bacterial DNA kit (Tiangen Biotech, China). The pair of primers used for polymerase chain reaction (PCR) amplification and sequencing were Coryn-1 (GCG GCT ACG TAA AGT CC) and Coryn-2 (CCG CCG GAGCCG TTC AT). For PCR amplification, the protocol detailed by Sierra et al. was followed [5]. The PCR products were sequenced with the same primers as those used in PCR amplification on ABI 3730XL DNA Analyzer (Applied Biosystems, USA). Additionally, the sequence of *C. striatum* ATCC6940 was used as the control for sequence comparison among different clinical *C. striatum* isolates.

## Results

### Characterization of the isolates

From the 410 isolates analyzed in this study, 77.8% (319/410) were collected from hospital A, 10.0% (41/410) from hospital B, and 12.2% (50/410) from hospital C. Most of the *C. striatum* strains (88.3%; 362/410) were isolated from sputum (Table 1). The average age of the patients was 63 years, and male and female patients accounted for 71.2% (292/410) and 28.8% (118/410), respectively.

**Table 1:** Demographic and clinical features of patients (n = 410)

Variable	Number (%)
Age (years)	
< 40	6.6 (27/410)
40–59	33.4 (137/410)
60–79	44.1 (181/410)
≥ 80	15.9 (65/410)
Gender	
Female	28.8 (118/410)
Male	71.2 (292/410)
Specimens	
Sputum	88.3 (362/410)
Wound discharge	4.1 (17/410)
BALF	1.5 (6/410)
Whole blood	1.2 (5/410)
Pus	1.0 (4/410)
Urine	0.7 (3/410)
Central venous catheters	0.7 (3/410)
Nasopharyngeal swab	0.7 (3/410)
Drainage	0.7 (3/410)
Hydrothorax and ascites	0.5 (2/410)
Cerebrospinal fluid	0.5 (2/410)

*BALF* Bronchoalveolar lavage fluid

### Antibiotic susceptibility testing

The antibiotic susceptibility testing results showed that all strains were sensitive to vancomycin, linezolid, and daptomycin and resistant to penicillin, cefepime, ciprofloxacin, and moxifloxacin. The total resistance rates of 410 *C. striatum* isolates to imipenem, erythromycin, clindamycin, tetracycline, gentamycin, sulfamethoxazole, and trimethoprim were 90.7% (372/410), 98.8 (405/410), 98.5 (404/410), 70.5 (289/410), 53.2 (218/410), and 98.0 (402/410), respectively (Table 2).

**Table 2:** Antibiotics susceptibility profiles of 410 *C. striatum* strains

Antibiotics	MIC (μg/ml)			Percentage of resistant isolates, % (n/410)
	MIC_50_	MIC_90_	Range	
Penicillin	≥ 8	> 64	≤ 1, ≥ 4	100 (410/410)
Cefepime	≥ 8	> 64	≤ 1, ≥ 4	100 (410/410)
Imipenem	≥ 32	> 64	≤ 4, ≥ 16	90.7 (372/410)
Ciprofloxacin	≥ 32	64	≤ 1, ≥ 4	100.0 (410/410)
Moxifloxacin	8	16	≤ 0.5, > 0.5	100.0 (410/410)^a^
Erythromycin	32	64	≤ 0.5, ≥ 2	98.8 (405/410)
Clindamycin	16	> 32	≤ 0.5, ≥ 4	98.5 (404/410)
Tetracycline	≥ 32	> 64	≤ 4, ≥ 16	70.5 (289/410)
Gentamycin	8	≥ 32	≤ 4, ≥ 16	53.2 (218/410)
Sulfamethoxazole and trimethoprim	≥ 8/152	≥ 8/152	≤ 2/38, ≥ 4/76	98.0 (402/410)
Linezolid	< 0.5	< 0.5	≤ 2	0 (0.0)
Daptomycin	< 0.5	< 0.5	≤ 1	0 (0.0)
Vancomycin	< 0.5	< 0.5	≤ 2	0 (0.0)

^a^Based on EUCAST breakpoint for Corynebacterium spp

Based on the susceptibility testing results, 410 *C. striatum* strains can be classified into 12 resistance biotypes, designated as patterns R1–R12. Among the isolotes, those with resistance to erythromycin, clindamycin, imipenem, tetracycline, and gentamycin were classified to be nonsusceptible as they were resistant or intermediate to the five types of antibiotics tested in this study. Among these, three dominant biotypes (R1, R2, and R3) were identified, which were resistant or intermediate to most antibiotics tested in this study, except for vancomycin, linezolid, and daptomycin. The resistance features of the 12 resistant biotypes diversely changed (Table 3). In total, 319 isolates collected from hospital A belonged to 12 resistance biotypes, and most of the isolates belonged to R1 (46.1%; 147/319), R2 (24.5%; 78/319), and R3 (23.5%; 75/319). In addition, 41 isolates collected from hospital B were divided into eight resistance biotypes, and 56.1% (23/41) and 17.1% of the isolates (7/41) belonged to R1 and R2, respectively. Finally, 50 isolates collected from hospital C were classified into four resistance biotypes, and 48.0% (24/50) and 36.0% of the isolates (18/50) belonged to R1 and R3, respectively.

**Table 3:** Resistance biotypes of 410 *C. striatum* strains

Resistance biotypes	No. of Isolates (n)	Antibiotics											
		VAN	DAP	LNZ	P	FEP	CIP	SXT	E	CLI	IPM	TE	GEN
R1	194	S	S	S	R	R	R	R	R	I/R	I/R	I/R	I/R
R2	86	S	S	S	R	R	R	R	R	I/R	R	I/R	S
R3	95	S	S	S	R	R	R	R	R	R	I/R	S	I/R
R4	17	S	S	S	R	R	R	R	R	R	S	R	R
R5	6	S	S	S	R	R	R	R	I/R	R	R	S	S
R6	2	S	S	S	R	R	R	S	R	I/R	R	S	S
R7	2	S	S	S	R	R	R	S	R	R	R	R	I
R8	2	S	S	S	R	R	R	S	R	R	R	R	S
R9	2	S	S	S	R	R	R	R	S	S	R	I/R	S
R10	2	S	S	S	R	R	R	S	R	R	R	S	I
R11	1	S	S	S	R	R	R	R	S	R	R	R	R
R12	1	S	S	S	R	R	R	R	R	R	S	S	R

*VAN* vancomycin, *DAP* daptomycin, *LNZ* linezolid, *P* penicillin, *FEP* cefepime, *CIP* ciprofloxacin, *SXT* sulfamethoxazole and trimethoprim, *E* erythromycin, *CLI* clindamycin, *IPM* imipenem, *TE* tetracycline, *GEN* gentamycin

### Gene sequencing

Single-site and double-site mutations within the QRDRs of *gyrA* were observed among all 410 *C. striatum* strains, and eight mutations were observed in this study (Table 4). All isolates showed mutations in codon 87; 97.3% of the isolates (399/410) had double mutations in codons 87 and 91, whereas only 2.7% isolates (11/410) had a single mutation in codon 87. Ser-87 to Phe-87 and Asp-91 to Ala-91 double mutations in *C. striatum* accounted for 72.2% (296/410) of the isolates. Meanwhile, four new mutations in *gyrA* were found in this study, including Ser-87 to Tyr-87 and Asp-91 to Ala-91 (double mutation, 101 isolates), Ser-87 to Val-87 and Asp-91 to Gly-91 (double mutation, one isolate), Ser-87 to Val-87 and Asp-91 to Ala-91 (double mutation, one isolate), and Ser-87 to Ile-87 (single mutation, one isolate). Isolates with double mutations (96.5%; 385/399) in *gyrA* showed high minimum inhibitory concentration (MIC) of ≥ 32 µg/mL to ciprofloxacin, whereas 72.7% (8/11) of the *C. striatum* strains with a single mutation in *gyrA* presented with high MIC of ≥ 32 µg/mL to ciprofloxacin. For five quinolone-sensitive *C. striatum* clinical isolates collected in this study, no mutation in *gyrA* was identified.

**Table 4:** Mutation modes of QRDR region in *gyrA* gene among 410 *C. striatum* isolates

No. of isolates	Mutations of QRDRs within *gyrA* gene	
	Codon 87	Codon 91
296	S (Ser) → F (Phe)	D (Asp) → A (Ala)
101	S (Ser) → Y (Tyr)	D (Asp) → A (Ala)
1	S (Ser) → F (Phe)	–
4	S (Ser) → V (Val)	–
5	S (Ser) → Y (Tyr)	–
1	S (Ser) → I (Ile)	–
1	S (Ser) → V (Val)	D (Asp) → G (Gly)
1	S (Ser) → V (Val)	D (Asp) → A (Ala)

### PFGE

**Fig. 1:**
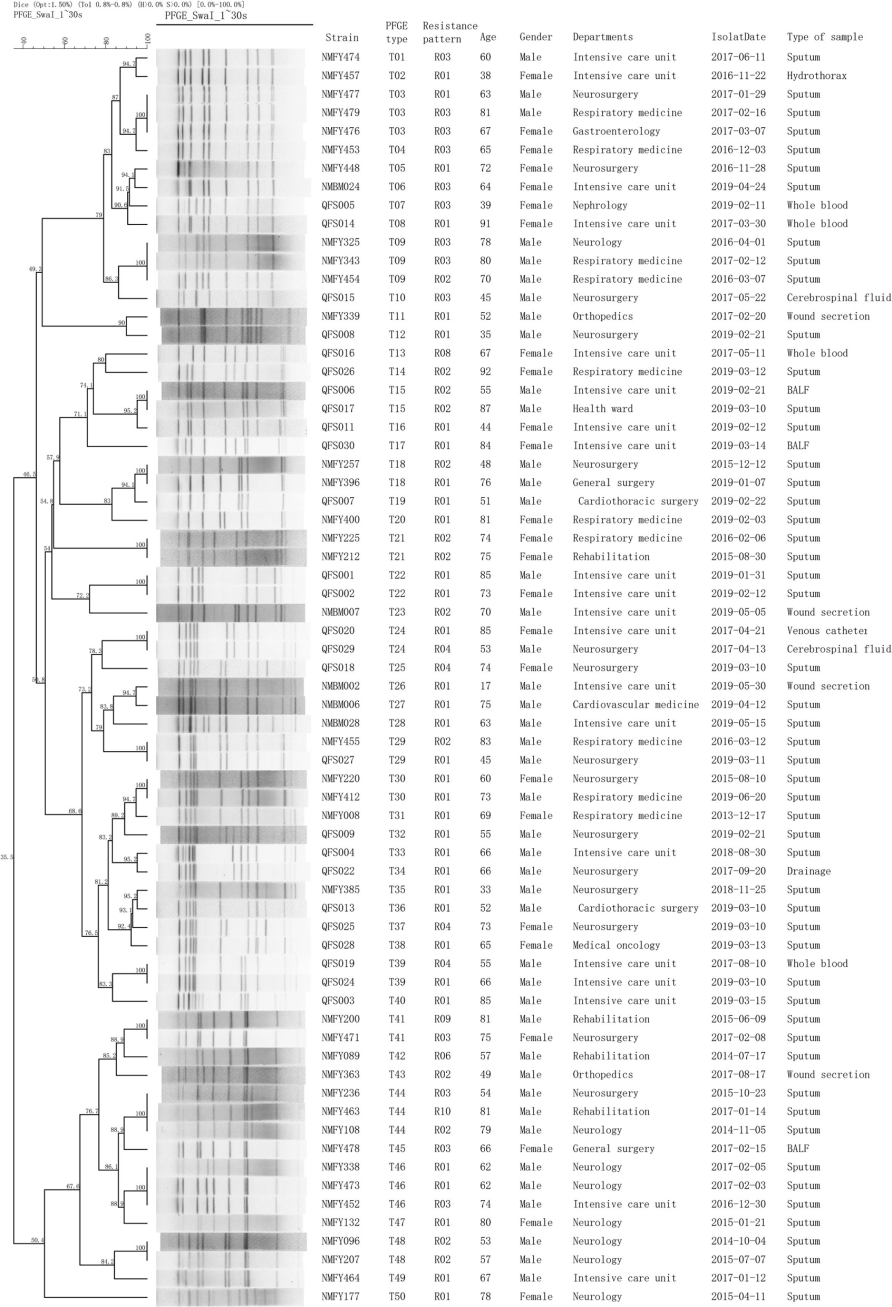
Molecular characterization of 101 *C. striatum* strains with different resistance biotypes. For each types of T02, T06, T20 and T27, only the PFGE gel of one representative isolate were presented here. T02 clone, T06 clone, T20 clone, and T27 clone were composed by 12, 6, 4 and 15 isolates, respectively

**Fig. 2:**
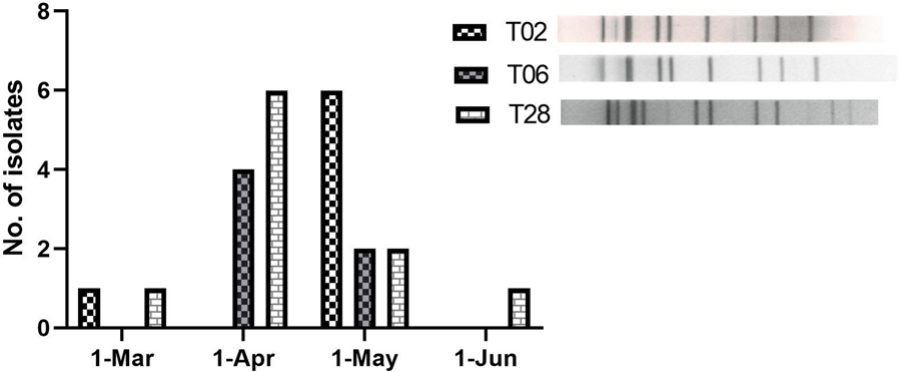
Prevalence of three dominant clones (T02, T06 and T27) among the patients admitted to ICU during March to June, 2019 in hospital C

A total of 101 *C. striatum* strains with different resistance biotypes were randomly selected for PFGE typing, and 50 PFGE types were identified, among which types T02 (15 isolates), T06 (6 isolates), and T28 (12 isolates) were dominant clones that accounted for 14.9% (15/101), 5.9% (6/101), and 11.9% (12/101), respectively. The isolates belonged to the clones with similar antibiotic resistance features and were multidrug resistant. These three clones were mainly isolated from patients admitted to the neurosurgery unit and intensive care unit (ICU) (Fig. 1). The T02 clone prevailed in hospital A (40.00%; 6/15) and hospital C (60.00%; 9/15), whereas the T06 and T28 clones were only isolated from the patients admitted to hospital C (18/18; 100.0%). The T02 clone showed a long-term persistence in hospital A from 2016 to 2018, and an outbreak was observed in the ICU of hospital C from May 12 to May 19, 2019. The other two outbreaks of the T06 and T28 clones also emerged in the ICU of hospital C from April to May 2019 (Fig. 2). Furthermore, three dominant clones showed high levels of ciprofloxacin or moxifloxacin resistance, and the MICs of moxifloxacin of all isolates were significantly lower than those of ciprofloxacin. The moxifloxacin MIC of the *C. striatum* strains belonging to the T02 clones was ≥ 8 µg/mL, which was higher than that of the strains belonging to the T06 and T28 clones. Double mutations in codons 87 (S → F/Y) and 91 (D → A) of *gyrA* were identified in these three dominant clones (Table 5).

**Table 5:** Characterization of quinolone resistance of three dominant *C. striatum* clones

Clones	Ciprofloxacin (µg/mL)		Moxifloxacin (µg/mL)		Sequence of *gyrA* gene (aa)	
	MICs	Sensivity (≤ 1, ≥ 4)	MICs	Sensivity (≤ 0.5, > 0.5)	87-S	91-D
T28	≥ 32	R	≥ 4	R	F	A
T06	≥ 32	R	≥ 4	R	Y	A
T02	≥ 32	R	≥ 8	R	Y	A

In the three nosocomial outbreaks caused by the three clones of *C. striatum*, 33 patients were involved and 12.1% (4/33) of the patients died. The average age of patients and length of hospital stay were 58 years and 30 d, respectively. Moreover, 97% (32/33) of the patients were exposed to different antibiotics 2 weeks before *C. striatum* isolation, and most of these patients had different comorbidities, including cerebrovascular events (39.4%; 13/33), chronic obstructive pulmonary disease (36.4%; 12/33), and malignant diseases (21.1%; 7/33) (Table 6).

**Table 6:** Descriptive characteristics of 33 patients involved in three nosocomial outbreaks

Variable	Number (%)
Length of hospital stay (days)	
≤ 7	6.1 (2/33)
8–14	9.1 (3/33)
15–21	24.2 (8/33)
> 21	60.6 (20/33)
Age (years)	
< 40	6.1 (2/33)
40–59	33.3 (11/33)
60–79	51.5 (17/33)
≥ 80	9.1 (3/33)
Outcome of the patients	
Death	12.1 (4/33)
Antibiotic intake^a^	
Cephalosporins	42.4 (14/33)
Carbapenem	24.2 (8/33)
β-lactam/β-lactamase inhibitor combinations	36.4 (12/33)
Quinolones	15.2 (5/33)
Glycopeptides	12.1 (4/33)
Aminoglycosides	0 (0/33)
Macrolides	0 (0/33)
Lincosamides	0 (0/33)
Trimethoprim-Sulfamethoxazole	0 (0/33)
Comorbid diseases	
Cerebrovascular event	39.4 (13/33)
Chronic obstructive pulmonary disease	36.4 (12/33)
Malignant diseases	21.1 (7/33)
Diabetes mellitus	9.1 (3/33)
Chronic renal failure	6.1 (2/33)
Heart failure	3.0 (1/33)

^a^The antibiotic intake was calculated within two weeks before *C. striatum* isolation

## Discussion

### Resistance features of *C. striatum* isolates

In this study, most of the 410 *C. Striatum* strains were isolated from lower respiratory tract samples (93.2%; 382/410). Meanwhile, five *C. striatum* strains were isolated from whole blood samples and one strain was repeatedly isolated from an immunosuppressed patient with infective endocarditis. Consistent with the results of previous investigations [6, 16], the *C. striatum* strains detected in this study were sensitive to vancomycin and linezolid. By contrast, all isolates were ciprofloxacin-resistant, and most had high MICs (≥ 32 µg/mL) to ciprofloxacin. Three dominant resistance biotypes were identified in this study, which accounted for 91.5% (375/410), and the strains were resistant to penicillin, cefepime, ciprofloxacin, and erythromycin. Although the isolates from the three hospitals were categorized into 12 resistant biotypes, no significant difference in geographical distribution was observed. Daptomycin resistance in *C. striatum* has been reported in previous studies [17, 18]; however, no daptomycin-resistant isolate was identified in this present study.

### Mechanism of quinolone resistance in *C. striatum* isolates

The level of quinolone resistance depended on the type of amino substitution; isolates with double mutations on codons 87 and 91 showed a higher level of quinolone resistance than those with a single mutation on codon 87 [5, 16]. In this study, most of the quinolone-resistant *C. striatum* isolates were confirmed to have double mutations in *gyrA* (codons 87 and 91). However, not all isolates presented with MIC of ≥ 32 µg/mL to ciprofloxacin. By contrast, a single mutation in *gyrA* (codon 87) was detected in 11 *C. striatum* isolates, and the MICs of 72.7% (8/11) isolates to ciprofloxacin were ≥ 32 µg/mL. The significant relationship between amino mutation patterns and resistance levels to quinolones was not observed in this study. Furthermore, four new mutation patterns in *C. striatum* were found in this study, including three double mutations in codons 87 and 91 of *gyrA* and a single mutation in codon 87 of *gyrA*. Two kinds of new mutations, the double mutation pattern (87-S → Y, 91-D → A) and the single mutation pattern (87-S → I), were previously found in *C. urealyticum* and *C. jeikeium* [4]. To the best of our knowledge, the remaining two types of newly detected mutation patterns in *C. striatum* have not been reported previously, which had double mutations in codon 87 and 91. In particular, isolates with the double mutation pattern (87-S → Y, 91-D → A) accounted for 24.6% (101/410). Further, Sierra et al. [5] found that some *C. striatum* strains with a single mutation in codon 87 were ciprofloxacin-resistant but sensitive to moxifloxacin, suggesting that other resistance mechanisms contribute to moxifloxacin resistance in *C. striatum*. However, no moxifloxacin-sensitive strain was detected in this study, and a higher level of resistance of *C. striatum* to moxifloxacin was observed. The MIC_90_ of the 410 *C. striatum* isolates to moxifloxacin was high, i.e., up to 8 µg/mL, indicating severe quinolone resistance in *C. striatum* in these hospitals. Unfortunately, quinolones appear to exert double effects on *C. striatum*, which could both induce its resistance to quinolones and promote *C. striatum* acquisition among susceptible patients [3, 9]. Cumulatively, preventing quinolone exposure appears to be the most efficient way to control *C. striatum* infection or prevent the development of resistance to quinolones.

### Relationship between PFGE typing and antimicrobial resistance

To further verify whether some dominant clones could prevail in a hospital environment, the PFGE typing method was employed, which is considered the gold standard method for the epidemiological investigation of important hospital-acquired bacterial pathogens [19]. A previous study revealed that the multidrug resistance phenotype of *C. striatum* is associated with specific PFGE types [20]. However, another study presented controversial results that the resistance phenotype was not related to specific clones [9], consistent with the findings of this present study. Because most of the strains detected in this study were multidrug resistant, the corresponding relationship between the PFGE genotypes and resistance biotypes could not be identified based on the study results.

### Nosocomial transmission of the dominant *C. striatum* clones

Consistent with the findings of this study, Baio et al. [20] reported that some dominant *C. striatum* clones could rapidly spread among inpatients and that *C. striatum* could transmit from person to person via healthcare personnel or the medical environment [21, 22]. Three dominant clones were identified in this study, which persisted in the several wards of hospital A from 2016 to 2018. This suggests a strong fitness ability of *C. striatum* among susceptible patients within the hospital environment. Unfortunately, based on the current data, it was difficult to confirm whether the dominant clones belonged to the same origin in these three hospitals as they are located at long distances. Further, three outbreaks were observed among patients admitted to the ICU of hospital C in 2019, resulting from the T02, T06, and T28 clones, respectively. Of note, the three clones coexisted among different patients in the ICU of hospital C from April to June 2019, indicating a multisource feature and rapid spread of *C. striatum*. Therefore, more efficient measures for infection control should be implemented to better controlling the transmission of *C. striatum*. Moreover, 33 patients were confirmed to be involved in these three nosocomial outbreaks; most of these patients had at least one comorbidity and 97% (32/33) were exposed to at least one broad-spectrum antibiotic 2 weeks before *C. striatum* isolation. This finding implies that for special hospital units with critically ill patients, more attention should be paid when *C. striatum* strains are isolated.

A recent report revealed that some dominant *C. striatum* clones showed resistance to some widely used biocides at different levels, including high-level disinfectants (such as glutaraldehyde) [23]. Therefore, the sensitivity of dominant *C. striatum* isolates from different hospitals to commonly used biocides should be further evaluated, and the selective use of effective biocides should be considered. In this study, 50 PFGE genotypes were identified among 101 *C. striatum* isolates, and high genotyping diversity was observed in the strains isolated from the same hospital, particularly those isolated from hospitals A and B. This suggests that multidrug *C. striatum* isolates originate from diverse origins and more dominant clones are selected under suitable circumstances, such as an increasing number of susceptible patients and nonrational application of broad-spectrum antibiotics.

## Limitations

The limitations of this study are indicated as follows. Only 101 *C. striatum* isolates were genotyped using the PFGE method owing to the limitation of the expenditure. The actual classification and distribution of different clones among different wards or patients may lack accuracy owing to selection bias. Therefore, dominant clones and nosocomial outbreaks may have been underestimated based on these data. Furthermore, a limited number of isolates were obtained from hospitals B and C, and only the isolates from 2019 were eventually analyzed in this study. Therefore, the actual resistant phenotypes of *C. striatum* isolates and prevalence potential of dominant clones within these two hospitals need to be further explored.

## Conclusions

Most *C. striatum* clinical strains analyzed in this study showed multidrug resistance, particularly to quinolones. The diversity of mutation in the *gyrA* of *C. striatum* is reported for the first time in this study. The strict restriction of quinolone use and prevention of its selective pressure on *C. striatum* may be the most effective way to minimize *C. striatum* colonization or infection and suppress the development of its resistance to quinolones. Dominant clones should be paid more attention in future infection control strategies as they can persist in hospitals for a long period and widely spread among susceptible patients.

## Data Availability

None.

## Abbreviations

QRDRs: Quinolone Resistance-Determining Regions
PFGE: Pulsed-field gel electrophoresis
PCR: Polymerase chain reaction
MIC: Minimum inhibitory concentration
ICU: Intensive care unit
